# Methylation of *FKBP5* and *SLC6A4* in Relation to Treatment Response to Mindfulness Based Stress Reduction for Posttraumatic Stress Disorder

**DOI:** 10.3389/fpsyt.2018.00418

**Published:** 2018-09-18

**Authors:** Jeffrey R. Bishop, Adam M. Lee, Lauren J. Mills, Paul D. Thuras, Seenae Eum, Doris Clancy, Christopher R. Erbes, Melissa A. Polusny, Gregory J. Lamberty, Kelvin O. Lim

**Affiliations:** ^1^Department of Experimental and Clinical Pharmacology, University of Minnesota College of Pharmacy, Minneapolis, MN, United States; ^2^Department of Psychiatry, University of Minnesota Medical School, Minneapolis, MN, United States; ^3^University of Minnesota Supercomputing Institute, Minneapolis, MN, United States; ^4^Minneapolis Veterans Affairs Health Care System, Minneapolis, MN, United States; ^5^Defense Veterans Brain Injury Center, Minneapolis, MN, United States

**Keywords:** PTSD, meditation, MBSR, treatment response, epigenetics, DNA methylation, FKBP5, SLC6A4

## Abstract

Mindfulness Based Stress Reduction (MBSR) is an effective non-pharmacologic treatment for veterans with PTSD. Extensive work has identified epigenetic factors related to PTSD disease risk and pathophysiology, but how these factors influence treatment response is unclear. Serotonin signaling and hypothalamic-pituitary-adrenal (HPA) axis functioning may be perturbed in PTSD and are molecular pathways targeted by PTSD treatments. To identify potential biomarkers for treatment response, we utilized genomic DNA isolated from peripheral blood samples from veterans with PTSD who were responders (*n* = 11) or non-responders (*n* = 11) to MBSR as part of a clinical trial. We assessed methylation levels at CpG sites in regions of the serotonin transporter (*SLC6A4*) previously associated with expression and depression outcomes, as well as the Intron 7 region of the FK506 binding protein 5 (*FKBP5*) containing known glucocorticoid response elements suggested to regulate this gene. Selected subjects were matched across MBSR responder status by baseline symptoms, age, sex, current smoking status, and current antidepressant use. Percent methylation was compared between responders and non-responders at baseline (pre-MBSR treatment). Additionally, percent change in methylation from baseline to post-treatment was compared between responders and non-responders. There was a significant time x responder group interaction for methylation in *FKBP5* intron 7 bin 2 [*F*_(1, 19)_ = 7.492, p = 0.013] whereby responders had an increase in methylation and non-responders had a decrease in methylation from before to after treatment in this region. Analyses of the three CpG sites within bin 2 revealed a significant time x responder group interaction for CpG_35558513 [*F*_(1, 19)_ = 5.551, *p* = 0.029] which resides in a known glucocorticoid response element (GRE). Increases in *FKBP5* methylation after treatment in responders as compared to decreases in non-responders suggest that effective meditation intervention may be associated with stress-related pathways at the molecular level. These preliminary findings suggest that DNA methylation signatures within *FKBP5* are potential indicators of response to meditation treatment in PTSD and require validation in larger cohorts.

## Introduction

Post-traumatic stress disorder (PTSD) is a debilitating condition with a lifetime prevalence of 8–19% in combat veterans (1, 2). High rates of chronicity, poor quality of life, and severe functional impairments associated with PTSD result in ~$3 billion of lost productivity each year (3). Sadly, only 20–30% of patients experience remission of PTSD symptoms from pharmacologic and nonpharmacologic treatment interventions (4, 5). Extensive work has identified genetic and epigenetic factors related to PTSD disease risk and pathophysiology, but there remains a critical need to leverage this knowledge to develop new treatments and also improve response to existing therapies.

Mindfulness-based stress reduction (MBSR) is a meditation approach to promote relaxation and achieve a heightened sense of well-being (6) which has gained increasing evidence for efficacy in the treatment of PTSD (7–10). MBSR brings together mindfulness meditation and yoga. Mindfulness cultivates a greater awareness of the mind and body, unconscious thoughts, feelings, and behaviors that can undermine emotional, physical, and spiritual health. MBSR appears to be a viable treatment option for PTSD, with a 49% response rate in a recent trial of patients with long-standing and symptomatic illness who had not experienced extensive relief with medications (9). The exact mechanistic underpinnings of MBSR efficacy are not known. Reductions in markers of stress reactivity, such as cortisol have been observed as a result of a mindfulness program for PTSD (11), implicating effects through the hypothalamic-pituitary adrenal (HPA) axis. Preliminary electroencephalogram (EEG) and imaging studies additionally suggest that meditation may influence brain activity and structure in regions associated with neurotransmitter synthesis and release (12, 13).

Genes involved with monoamine and HPA axis function have been extensively examined in epigenetic and genetic studies of PTSD risk, and separately in studies of disease risk and response to treatments for mood disorders (14–17). Two of the most commonly characterized genes in this regard are the serotonin transporter (*SLC6A4*) and FK506 binding protein 5 (*FKBP5)*. *SLC6A4* encodes the serotonin transporter (5HTT) which regulates serotonin reuptake from the synaptic cleft. Disruption of 5HTT function can be caused by epigenetic modification of the promoter region of this gene whereby increased methylation decreases 5HTT expression (18). Altered expression as a result of methylation may influence the likelihood of an individual developing PTSD after traumatic events (19). Additionally, hypomethylation of the serotonin transporter may decrease response to antidepressant treatment for depression (16). To date, relationships between *SLC6A4* methylation and response to treatments for PTSD have not been examined.

*FKBP5* encodes the FK506 binding protein 5 which regulates the glucocorticoid receptor complex through binding and impeding its translocation to the nucleus (20). In this role, FKBP5 serves as part of the negative feedback loop regulating glucocorticoid activity and acute stress response. FKBP5 is activated by glucocorticoid interactions with intronic regulatory glucocorticoid response elements (GREs) which increase transcription, and then feeds back to attenuate glucocorticoid signaling. This acute regulation of stress response occurs through a dynamic process of methylation/de-methylation (21–23). Traumatic childhood exposures are thought to imprint long-standing patterns of demethylation, resulting in chronically dysregulated feedback and risk for PTSD (20). Preliminary evidence in one small study suggests that *FKBP5* methylation patterns may change in veterans with PTSD who respond to exposure therapy (24).

Methylation influences biological responses to environmental stimuli and similarly, environmental exposures alter gene regulation through methylation changes. Thus, the examination of methylation patterns as biomarkers which may predispose an individual to respond to treatment or change as a result of treatment may reveal important mechanistic insights to help optimize interventions and develop new treatments. While gene expression and regulating factors may differ across cell and tissue types, peripheral DNA from blood may serve as an accessible source for DNA methylation biomarker studies. We conducted a pilot study investigating the methylation of *SLC6A4* and *FKBP5* genes before and after MBSR in veterans with PTSD who responded or did not respond to 9 weeks of treatment as defined by changes in scores on the PTSD Checklist rating scale. We hypothesized that decreased methylation in *SLC6A4* and *FKBP5* would be associated with better treatment response to MBSR.

## Materials and methods

### Study design

We conducted a case-control candidate gene study investigating relationships between *SLC6A4* and *FKBP5* methylation with responders (*n* = 11) and non-responders (*n* = 11) to MBSR in veterans with PTSD. Comparisons in methylation measures at baseline and then changes after 9 weeks of MBSR were examined between responders and non-responders.

### Participants

Participants (*n* = 22) represent a subsample of a larger clinical trial (NCT01548742) of *n* = 116 veterans recruited at the Minneapolis Veterans Affairs Health Care System from March 2012 to December 2013 which compared MBSR to Present Centered Group Therapy (PCGT) for the treatment of PTSD (9). All participants included in this pilot methylation study were Caucasian. Participants were at least 18 years of age, met the *Diagnostic and Statistical Manual of Mental Disorders* (Fourth Edition) (*DSM-IV*) (25) diagnostic criteria for PTSD based on the Clinician-Administered PTSD Scale (CAPS) (26) and, if taking psychoactive medication, were on a stable regimen for 4 or more weeks prior to study enrollment. Participants were excluded if they (a) reported current suicidal or homicidal ideation; (b) met DSM-IV criteria for current substance abuse or dependence (except nicotine or caffeine) per self-reported answers on the diagnostic interview or (c) met DSM-IV criteria for a psychotic disorder; or (d) were diagnosed with severe cognitive impairment, severe traumatic brain injury, or medical illness that could interfere with treatment; or (e) were unable to comprehend or communicate in English; or (f) were unwilling to refrain from other active forms of psychotherapy during the study period. This study was carried out in accordance with the recommendations of Minneapolis VA Health Care System and University of Minnesota institutional review boards with written informed consent from all subjects. All subjects gave written informed consent in accordance with the Declaration of Helsinki. The protocol was approved by both the Minneapolis VA Health Care System and University of Minnesota institutional review boards. Participants for this pilot study were selected to compare specific methylation measures between responders and non-responders to MBSR to support larger scale methylation and genetic studies of this participant cohort. Responders and non-responders were defined based on whether or not participants achieved a reduction of 10 or more points on the PTSD Checklist (PCL) for symptom severity (27). Participants for inclusion needed to have clinical data and DNA available for baseline and follow-up time points. From this larger group of *n* = 51 participants receiving MBSR, Caucasian responders (*n* = 11) and non-responders (*n* = 11) were selected for matching baseline symptoms (baseline PCL total score), age, sex, current smoking status, and current antidepressant use. These and other clinical and demographic variables are included in Table 1.

**Table 1:** Baseline characteristics of responders and non-responders to MBSR.

**Characteristics**	**Responders (*n* = 11)** **Mean (*S.D*.) or *n* (%)**	**Non-responders (*n* = 11)** **Mean (*S.D*.) or *n* (%)**	**Test statistic**	***p*-value**
Age	60.4 (14.5) range: 33–79	58.2 (10.2) range: 40–69	*t* = 0.409, 20df	0.69
BMI	31.64 (4.178)	31.09 (7.816)	*t* = 0.204, 20df	0.84
**SEX**				
Male	9 (82%)	9 (82%)	X^2^ = 0.0, 1df	1.0
Female	2 (18%)	2 (18%)		
**RACE**				
White	11 (100%)	11 (100%)	X^2^ = 0.0, 1df	1.0
**SERVICE ERA**				
OEF/OIF	2 (18%)	1 (9%)	X^2^ = 2.41, 3df	0.49
Gulf War	0 (0%)	2 (18%)		
Vietnam War	7 (64%)	6 (55%)		
Other	2 (18%)	2 (18%)		
**SERVICE DURATION**				
<1 year	1 (9%)	1 (9%)	X^2^ = 2.8, 3df	0.42
1–2 years	4 (36%)	1 (9%)		
2–5 years	3 (27%)	6 (55%)		
>5 years	3 (27%)	3 (27%)		
Any Childhood Trauma (Y/N)	4 (36%)	2 (18%)	X^2^ = 0.92, 1df	0.34
Lifetime trauma (number events)	9.0 (3.2)	6.5 (3.2)	*t* = 1.78, 20df	0.09
PCL Total Score	63.2 (7.3)	61.5 (14.3)	*t* = 0.36, 20df	0.36
PCL Reexperiencing	17.73 (3.6)	17.64 (5.4)	*t* = 0.047, 20df	0.96
PCL Avoidance	26.45 (3.01)	25.91 (5.5)	*t* = 0.29, 20df	0.77
PCL Arousal	19.0 (4.2)	17.9 (4.9)	*t* = 0.56, 20df	0.58
PHQ9 number of symptoms endorsed	13.5 (5.5)	15.3 (5.8)	*t* = −0.71, 20df	0.48
Number of psychotropic medications	1.4 (0.5)	1.8 (0.9)	*t* = −1.494, 20df	0.15
Antidepressant user	10 (91%)	10 (91%)	X^2^ = 0.0, 1df	1.0
SSRI user	7 (64%)	6 (55%)	X^2^ = 0.19, 1df	0.66
SRI (SSRI or SNRI) user	10 (91%)	8 (73%)	X^2^ = 1.22, 1df	0.27
More than one antidepressant user	6 (55%)	6 (55%)	X^2^ = 0.0, 1df	1.0
**SMOKING STATUS**				
Never	2 (18%)	4 (36%)	X^2^ = 1.07, 2df	0.59
Current	3 (27%)	3 (27%)		
Former	6 (55%)	4 (36%)		

*BMI, Body Mass Index; OEF/OIF, Operation Enduring Freedom / Operation Iraqi Freedom; PCL, PTSD Checklist; PHQ9, Patient Health Questionnaire 9; SSRI, Selective Serotonin Reuptake Inhibitor; SNRI, Serotonin and norepinephrine reuptake inhibitor; X^2^, Pearson's chi-squared test; t, Student's t-test*.

### Assessments

The primary outcome measure was change in PTSD symptom severity over time as assessed by the PCL (28) at all assessment points. Diagnoses were based on DSM-IV criteria for PTSD, and a severity score was calculated by summing frequency and intensity scores for all 17 symptoms. The minimal clinically important difference (MCID) for PTSD symptom severity is a reduction of 10 or more points on the PCL and CAPS. Comorbid depression symptoms were assessed using the Patient Health Questionnaire 9 (PHQ-9) (29). Participants also reported treatment satisfaction at week 9 using a scale ranging from 1 to 4 with higher scales indicating greater satisfaction. Demographic information, including self-reported race/ethnicity, was collected at baseline. Mental health treatment history, self-reports of current and prior smoking status, and measures of early life trauma (physical/sexual/emotional abuse, emotional/physical neglect, and alcoholic environment) were extracted from VA electronic medical records.

### Intervention

The standard protocol for MBSR used for this study included an orientation to the program that incorporated PTSD psychoeducation and treatment rationale, followed by 7 weekly 2.5-h group sessions and a 6.5-h retreat, for a total of 9 sessions over the course of 9 weeks. The program teaches participants to attend to the present moment (immediate emotional and physical states, including discomfort) in a nonjudgmental and accepting way. Sessions involved didactic training and practice in 3 meditation techniques including (1) body scan, (2) sitting meditation, and (3) mindful yoga. Further details on the procedures and fidelity assessments for the MBSR and clinical ratings have been previously presented (9).

### Methylation studies

Blood samples were collected in EDTA-treated vacutainer tubes at baseline (pre-treatment) and at week 9 (post-treatment) for this study. DNA was extracted (ArchivePure™ DNA Kit) and assessed with standard picogreen and UV absorbance QC procedures. Genomic DNA was normalized to a concentration of 20 ng/μl and bisulfite converted using the EZ DNA Methylation Kit (Zymo Research) following the manufacturer's protocol.

**Figure 1:**
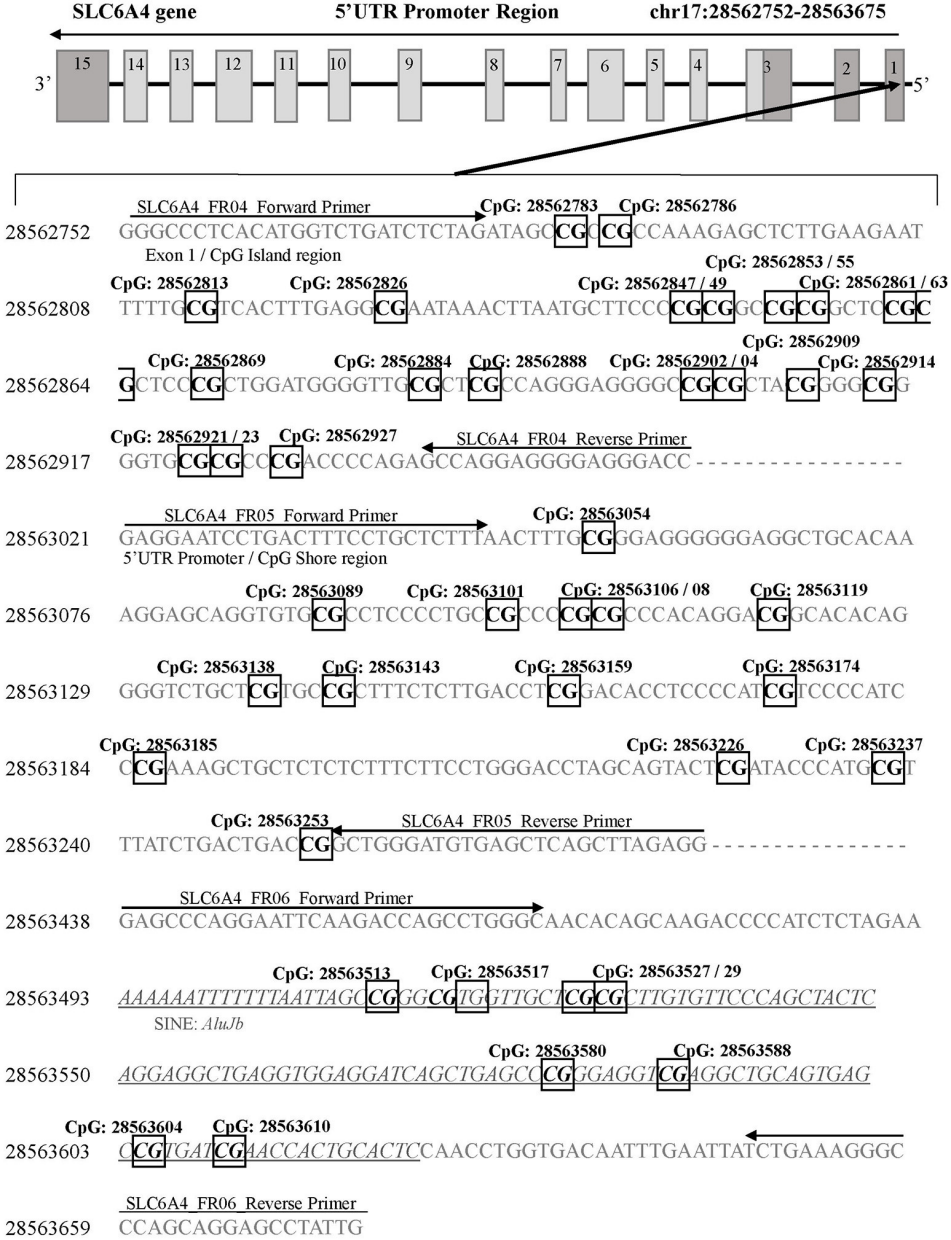
*SLC6A4* gene 5'UTR promoter region for DNA methylation detection. Three sets of primers were designed to cover the following: 1) the CpG island (SLC6A4_FR04; chr17: 28562752-28562954), 2) the CpG island shore (SLC6A4_FR05; chr17: 28563021-28563280), and 3) the *SLC6A4* upstream region containing a short interspersed nuclear element (AluJb) located between the *SLC6A4*-5HTTLPR and the CpG island (SLC6A4_FR06; chr17: 28563438-28563675). Primer binding locations are indicated by arrows. All detected CpG sites are boxed and in bold font. The AluJb region is indicated by underlined and italicized font. Coordinates for the *SLC6A4* gene region are based on Human Genome Build 37 (Human GRCh37/hg19).

**Figure 2:**
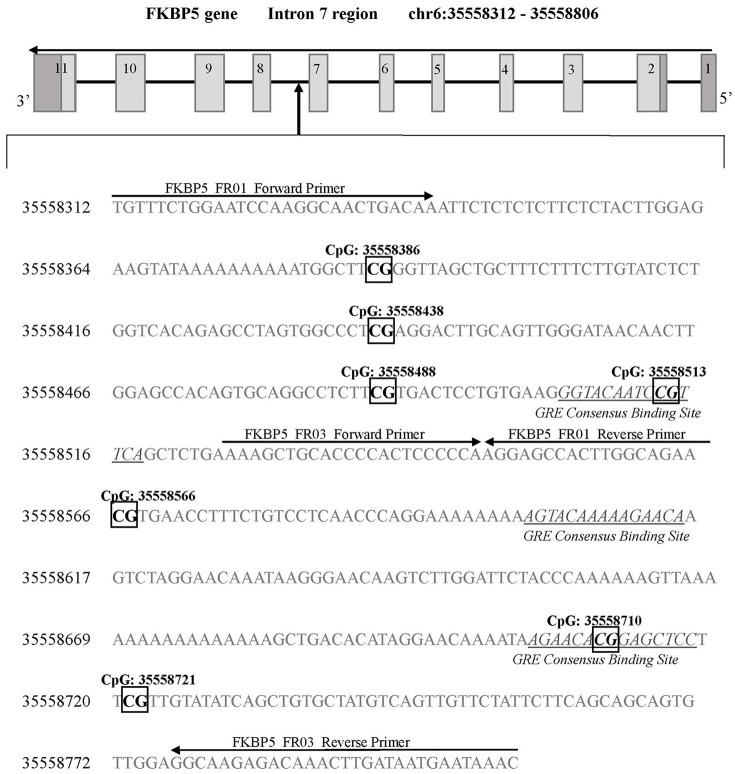
*FKBP5* gene intron 7 region for DNA methylation detection. Two sets of primers were designed to cover a 594 base pair region of intron 7 containing 7 CpG sites. FKBP5 intron 7 CpG sites are categorized into three bins as previously described (33): bin 1 contains CpG: 35558386 and CpG: 35558438, bin 2 contains CpG: 35558488, CpG: 35558513, and CpG: 35558566, and bin 3 contains CpG: 35558710 and CpG: 35558721. Primer binding locations are indicated by arrows. All detected CpG sites are boxed and in bold font. GREs are indicated by underlined and italicized font. Coordinates for the *FKBP5* intron 7 gene region are based on Human Genome Build 37 (Human GRCh37/hg19).

A next generation sequencing approach was used to examine methylation in regions of *SLC6A4* (Chr17: 28521337-28563020) and *FKBP5* (Chr6: 35541362-35696360). A total of five amplicons ranging from 203–254 bp were generated for selected areas of each gene. The regions selected for coverage examined 42 unique CpG sites (chr17:28562752-28563675) in the promoter region of *SLC6A4* previously associated with depression risk and symptoms (30, 31) (Figure 1) as well as 7 unique CpG sites in the *FKBP5* Intron 7 region (chr6:35558312-35558806) identified as a functional regulator of glucocorticoid signaling (32) (Figure 2). PCR primers for the amplification of *FKBP5* and *SLC6A4* gene target regions were designed to specifically amplify only bisulfite-modified DNA with consensus binding sites outside of known DNA SNPs with minor allele frequencies >0.5%. All PCR primers were tested with unmodified genomic DNA and showed no amplification to ensure specificity to bisulfite-treated DNA. Human methylated and unmethylated DNA controls (Zymo Research) served as positive and negative methylation controls and were used to ensure unbiased amplification of methylated or unmethylated, bisulfite-modified DNA. PCR primer and amplicon details are provided in Supplemental Table 1. Following primer optimization, sequencing was accomplished in three steps. First, independent PCR reactions were carried out for each bisulfite-modified DNA sample in high-throughput format. Second, all amplicons for each sample were pooled, and “barcoded” with a sample-specific sequence index using indexing primers. Finally, these pools were combined into a superpool for loading onto an Illumina MiSeq instrument, and run in paired-end 2 × 300-bp mode. Raw paired end reads were merged into single fragments using PEAR (33). Merged reads were aligned to the bisulfite human genome using WALT (34). Percent methylation and coverage at each CpG was calculated using the MethPipe utility methcounts (35). For each sample-amplicon pairing, 138–6,759 reads were generated, providing the ability to calculate CpG percent methylation with a precision of <0.1% at each site. Positive (known 100% methylated) and negative (known 0% methylated) controls were examined in duplicate for each amplicon to confirm expected percent methylation calling and to assess variance. Methylation variance between controls across all sequenced regions ranged from 0.52 to 0.75% and 1.15 to 1.63% in unmethylated and methylated controls, respectively. All samples and controls displayed >98% calculated bisulfite conversion rates. Utilizing the NCBI dbSNP database and Variation Viewer (https://www.ncbi.nlm.nih.gov/variation/view/), we identified all known variants within our target regions of *FKBP5* (chr6:35558312-35558806; total SNPs = 45) and *SLC6A4* (chr17:28562752-28563675; total SNPs = 81). Known variants identified within CpG sites of either *FKBP5* or *SLC6A4* target regions all contain reported minor allele frequencies of <0.01%. Due to the rarity of SNPs within CpG sites of our target regions, none of the 47 total CpG sites were excluded from analysis.

### Statistical analyses

Baseline differences between groups were examined using student's *t*-tests for continuously measured variables and χ^2^ statistics for categorical variables. Fisher's exact test was used when expected cell sizes were <5. To examine pre-treatment methylation differences, we compared % methylation in those who went on to respond versus those who did not respond to treatment at 9 weeks using analysis of covariance (ANCOVA) with lifetime trauma events used as covariate. Next we compared change in % methylation from before to after treatment in responders vs. non-responders using repeated measures analysis of variance (ANOVA) while accounting for lifetime trauma events. Due to the correlated nature of CpG sites within each gene, we employed a principal component data reduction approach for our primary analyses. Primary analyses examined mean % methylation in *FKBP5* CpG sites divided into three “bins” based on methylation patterns similar to previous investigations (32), and for *SLC6A4*, we identified one primary component comprised of *n* = 21 CpG sites in the CpG island region of the *SLC6A4* promoter similar to previous studies which have taken a PCA approach to data reduction in this gene (36). We were not powered to parse additional components of *SLC6A4* and thus focused our analyses on the primary component. *Post-hoc* examinations of individual CpG sites within bins were conducted for primary comparisons that yielded significance at the trend level or better (*p* < 0.10). Lastly, we examined methylation at baseline and changes after treatment in relation to PCL scores as a continuous measure using Pearson's correlation coefficients. Analyses were performed using SPSS (IBM Corp. Released 2013. IBM SPSS Statistics for Windows, Version 22.0. Armonk, NY: IBM Corp.).

## Results

Participants included in this analysis were predominantly male (*n* = 18, 82%), ranging from 33 to 79 years of age. Most participants (*n* = 13, 59%) were Vietnam era veterans with 68% having ≥2 years of service. Approximately 27% were current smokers, and 27% had a record of childhood trauma. Table 1 provides information on baseline demographics and clinical characteristics for the sample. There were no significant differences between responder and non-responder groups in demographic and clinical characteristics before treatment.

### Methylation of *SLC6A4*

#### Baseline (pre-treatment)

Methylation of the primary component of *SLC6A4* at baseline did not significantly differ between responders and non-responders. Similarly, when all participants were examined together regardless of responder status, *SLC6A4* methylation was not significantly correlated with PCL total scores at baseline (*r* = 0.324, *p* = 0.152).

#### Change from before to after MBSR treatment

There was no main effect of time for methylation changing in the primary component of *SLC6A4*. Similarly, we did not observe a significant correlation between methylation change and PCL change from before to after treatment (*r* = 0.216, *p* = 0.346).

### Methylation of *FKBP5*

#### Baseline (pre-treatment)

**Figure 3:**
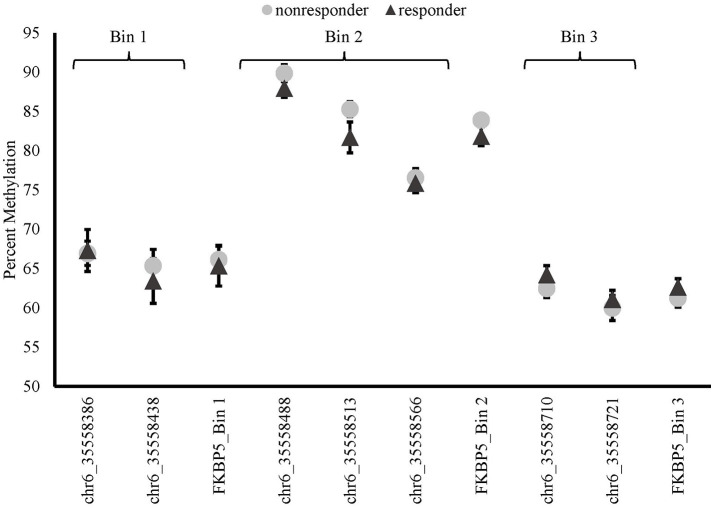
Methylation of *FKBP5* before treatment (baseline) in MBSR responders and non-responders. *FKBP5* intron 7 region contains 7 CpG sites located on chromosome 6 between nucleotide coordinates 35558312- 35558806.

There were no differences in baseline methylation identified between responders and non-responders for any of the *FKBP5* intron 7 bins examined (see Figure 3). *FKBP5* methylation was not significantly correlated with PCL change scores at baseline (bin1: *r* = −0.116, *p* = 0.616; bin2: *r* = −0.083, *p* = 0.721, bin3: *r* = –0.234, *p* = 0.308).

#### Change from before to after MBSR treatment

**Figure 4:**
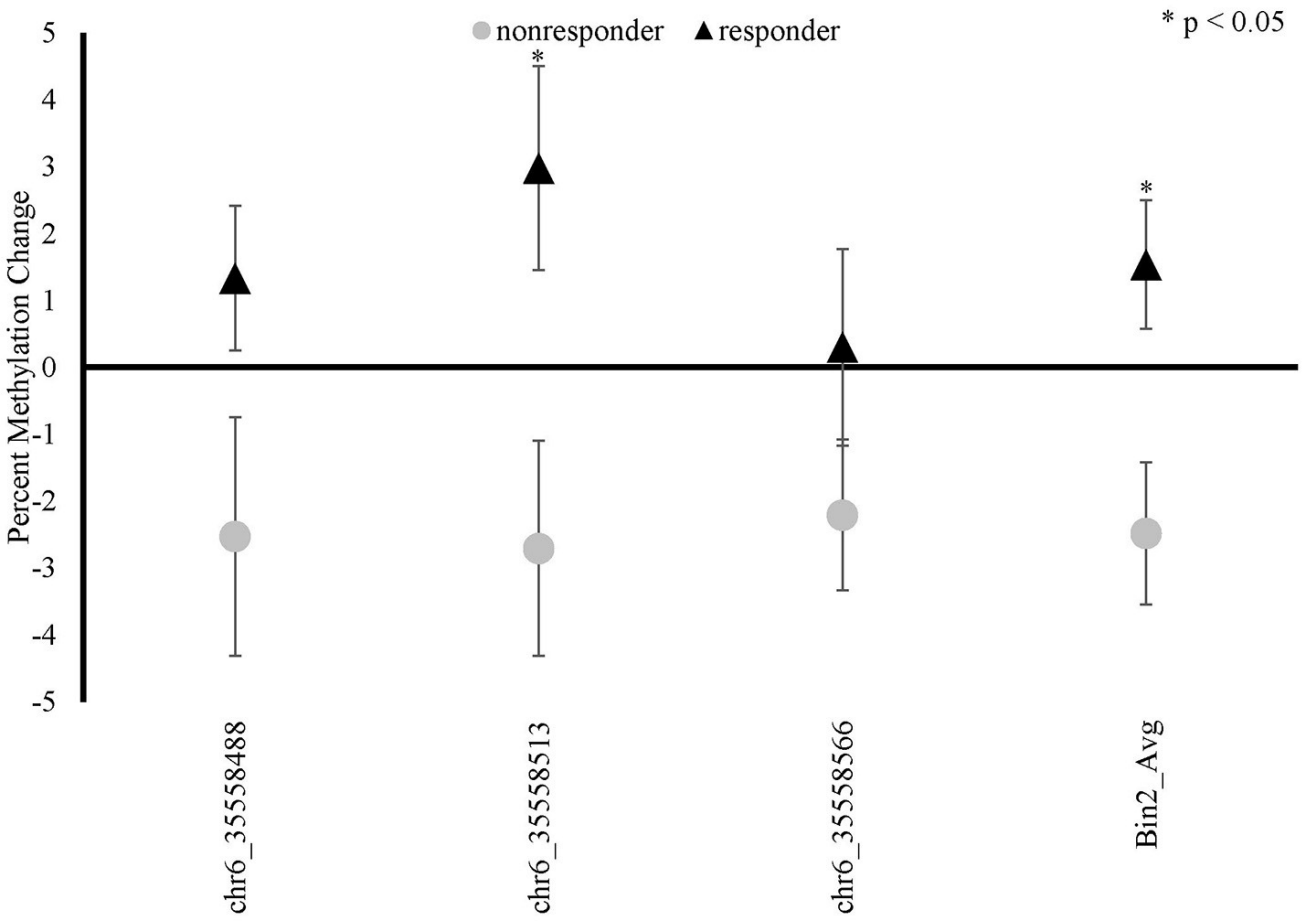
*FKBP5* methylation changes between baseline (before treatment) and 9-weeks (post-treatment) in MBSR responders and non-responders. *FKBP5* intron 7 region spans 7 CpG sites located between nucleotide positions 35558312- 35558806 on chromosome 6 (Human GRCh37/hg19). FKBP5 bin 2 showed a significant time x responder group interaction and contains three CpG sites.

**Figure 5:**
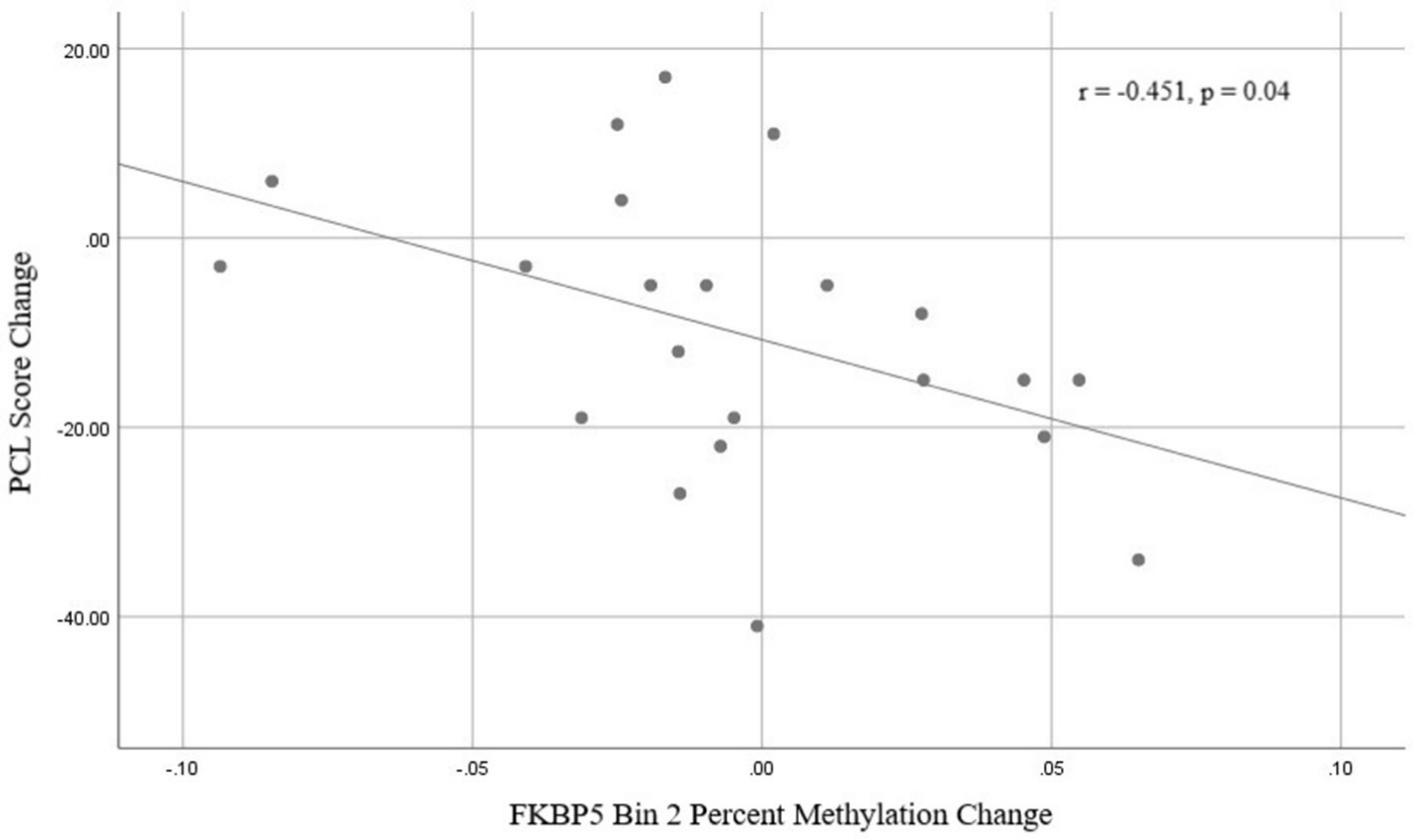
Correlation between *FKBP5* intron 7 bin 2 methylation change and PCL change scores from baseline (before treatment) and 9-weeks (post-treatment).

There was a significant time x responder group interaction for methylation in *FKBP5* intron 7 bin 2 [*F*_(1, 19)_ = 7.492, *p* = 0.013, Bonferroni adjusted *p* = 0.052; Figure 4 and Supplemental Table 2] whereby responders had an increase in methylation and non-responders had a decrease in methylation from before to after treatment in this region. Analyses of the three CpG sites of bin 2 revealed a significant time x responder group interaction for CpG_35558513 of bin 2 [*F*_(1, 19)_ = 5.551, *p* = 0.029; Figure 4 and Supplemental Table 2] which resides in a previously described GRE (32). *FKBP5* intron 7 bin2 methylation change and PCL change scores examined as continuous measures in all participants from before to after treatment were significantly correlated (*r* = −0.451, *p* = 0.04; Figure 5).

## Discussion

This is the first study to examine methylation relationships with treatment response to MBSR in veterans with PTSD who were carefully matched for other clinical and demographic variables. We identified methylation changes in relation to treatment response in a GRE of *FKBP5* intron 7 after 9 weeks of MBSR. These findings highlight the potential importance of meditation on genes which regulate HPA axis function.

### FKBP5

Analyses of *FKBP5* revealed increases in intron 7 methylation after treatment in responders while non-responders had decreases in methylation within a specific *FKBP5* CpG site of bin 2. FKBP5 is part of the stress feedback loop regulating glucocorticoid receptor function. As previously noted, FKBP5 upregulation feeds back to decrease glucocorticoid activity as part of a normal stress response, with demethylation usually resulting in increased expression. Previous studies of the intron 7 region of *FKBP5* and PTSD risk characterized allele-specific methylation patterns in relation to childhood trauma and the development of PTSD later in life (32). In that context, demethylation was associated with increased stress-related gene transcription and long-term dysregulation of stress regulation. To our knowledge ours is the first treatment study to examine changes in *FKBP5* intron 7 GREs in relation to improvement over time. This is significant as methylation in this region has been predominantly studied in the context of childhood trauma that was thought to impart chronic and irreversible methylation changes in this region.

To date, two studies have examined different regions of *FKBP5* methylation in response to psychotherapy, which were different non-pharmacological interventions than the MBSR approach employed in our protocol (24, 37). Both of these studies examined methylation in the *FKBP5* promoter region, which is different from the intron 7 focus of our study. Consistent with our study, baseline *FKBP5* methylation was not related to eventual responder status to either cognitive behavior therapy (CBT) in children with anxiety disorders (37) or exposure therapy (*n* = 8 responders or 8 non-responders) in veterans with PTSD (24). Additionally, consistent with our findings is that despite examining a different regulator region of *FKBP5* than our study (exon 1 promoter vs. intron 7 GRE in our study) both of these investigations showed that changes in methylation were related to better symptom response with corresponding measures of increased gene expression identified in the study of PTSD response to exposure therapy (24).

Our study further adds to the body of evidence suggesting that *FKBP5* methylation may be modified by non-pharmacological interventions and that regulatory regions (i.e., intron 7 GREs studied here) thought to represent relatively stable methylated regions are in fact malleable through therapies that may help patients regulate stress feedback. Collectively our findings as well as those of others are consistent with the hypothesis that non-pharmacologic interventions may facilitate stress reduction through the regulation of *FKBP5* to feedback on glucocorticoid hyperactivity to reduce stress.

### Serotonin transporter

In the CpG island region of the serotonin transporter prioritized for analysis in this study, we did not observe significant relationships with treatment response. To our knowledge, the only studies specifically examining baseline methylation in relation to treatment response have involved antidepressant medications for depression (16, 30). These two studies had conflicting results with respect to higher or lower pre-treatment methylation predicting better outcomes. Amidst a growing body of data establishing the deleterious influence of early life trauma on antidepressant response for major depression later in life (38) and the known influence of early life trauma on *SLC6A4* methylation (30), the relevance of these drug treatment studies for depression to our non-pharmacological intervention for PTSD is unclear. It is important to note that the serotonin transporter is generally expressed at lower levels in WBCs (as opposed to exclusively examining platelets), and thus perhaps not an optimal biomarker to study using this sampling methodology. Additionally, there are regions beyond the CpG island that may warrant future study. Our small sample size precluded us from parsing out additional components for analyses. The shore and AluJB regions near the promoter, as well as others may be relevant for future studies. Most (91%) of our participants were on stable antidepressant treatment as PTSD has a high rate of comorbid depression. Depression scores, rates of early life trauma, and medication exposures did not significantly differ in our responder and non-responder groups.

## Limitations

Our pilot study was small, designed to assess whether there was preliminary evidence to support larger investigations with more comprehensive methylation and genetic studies. As such, there are other genes (notably monoamine genes beyond *SLC6A4*) not assessed herein, that may be potentially informative. We were not powered to examine the influences of factors that are generally highly variable in larger sample sizes which include age, sex, race, smoking, medication exposures, trauma histories, genotype interactions, etc. However, recognizing this limitation, we carefully matched our responder and non-responder groups so that these factors did not differ or were equally represented in each responder group of our focused study sample. It is important to note that smoking and alcohol consumption have been reported to influence DNA methylation (39, 40). While the distribution of current, former, and non-smokers did not differ across responder and non-responders in our study, we are not able to unequivocally rule out the influence of smoking. Similarly, while none of the participants in our study met criteria for current alcohol (or other substance) use or abuse, a more detailed assessment of alcohol consumption may be informative. Our study was too small to examine the effects of known genotypes in *SLC6A4* (e.g., the 5HTTLPR) and *FKBP5* (e.g., rs1360780), which may interact with gene methylation to influence gene expression. Additionally, the *SLC6A4* results need to be interpreted with caution given the overall methylation levels observed in this gene were very low. This pilot study was not powered to control for multiple statistical comparisons. Consequently our findings require replication with larger and more diverse samples. The small sample size of our study and demographically homogeneous responder and non-responder groups may limit the broader generalizability of our methylation findings in relation to MBSR. However, we observed important consistencies with *FKBP5* findings with prior genetic studies examining different disease states or interventions, supporting the mechanistic importance of the findings observed here. Finally, it is important to note the limitations of using white blood cell DNA, where cell composition may influence the relative expression of genes. With respect to WBCs, the serotonin transporter is expressed at low levels, primarily in lymphocytes.

## Conclusions

We report herein the first study to examine pre- and post-treatment methylation patterns in relation to response to MBSR in veterans with long standing and symptomatic PTSD. Prior studies have suggested that *SLC6A4* or *FKBP5* methylation or genotypes which regulate gene expression influence the risk for developing PTSD (14, 19, 41). It is important to note, however, that these are not deterministic factors. Our study was comprised exclusively of veterans who had long-standing PTSD. Whether methylation or other genetic factors related to these genes were related to the development of PTSD in these individuals is unclear. Regardless of disease etiology, we identified significant changes between pre-treatment and post-treatment *FKBP5* methylation in responders and non-responders, highlighting the potential utility of methylation in this, and perhaps related genes, as biomarkers of treatment response. These findings add important mechanistic information related to treatment response that builds on a wealth of knowledge that exists about epigenetic and genetic relationships with PTSD disease risk.

## Author contributions

All authors contributed to the creation of the manuscript and approve of the final submitted version. All authors agree to be accountable for all aspects of the work in ensuring that questions related to the accuracy or integrity of any part of the work are appropriately investigated and resolved. JB study design/conception, clinical/experimental data analysis, interpretation of results. AL experimental design, experimental data generation. LM clinical and experimental data analysis, interpretation of results. SE data analysis and interpretation of results. DC clinical data acquisition and interpretation. PT clinical data acquisition and interpretation, clinical data analysis. CE clinical study design/conception, clinical data acquisition and interpretation. MP clinical study design/conception, clinical data acquisition and interpretation. GL clinical study design/conception, clinical data acquisition and interpretation. KL clinical study design/conception, clinical data interpretation, specimen acquisition.

### Conflict of interest statement

The authors declare that the research was conducted in the absence of any commercial or financial relationships that could be construed as a potential conflict of interest.

## Supplementary material

Supplemental Table 1 DNA methylation sequence amplicons and primers for *SLC6A4* and *FKBP5* genes. Coordinates are based on Human Genome Build 37 (Human GRCh37/hg19). All primers contained one of the following tagged sequences: (1) tcgtcggcagcgtcagatgtgtataagagacag- left primer, or (2) gtctcgtgggctcggagatgtgtataagagacag- right primer.

Supplemental Table 2 Adjusted means and 95% confidence intervals of methylation in *FKBP5* intron 7 bin2 and CpG_35558513 before and after treatment in MBSR responders and nonresponders.
