# Corrigendum: Methylation of *FKBP5* and *SLC6A4* in Relation to Treatment Response to Mindfulness Based Stress Reduction for Posttraumatic Stress Disorder

**DOI:** 10.3389/fpsyt.2021.642245

**Published:** 2021-03-04

**Authors:** Jeffrey R. Bishop, Adam M. Lee, Lauren J. Mills, Paul D. Thuras, Seenae Eum, Doris Clancy, Christopher R. Erbes, Melissa A. Polusny, Gregory J. Lamberty, Kelvin O. Lim

**Affiliations:** ^1^Department of Experimental and Clinical Pharmacology, University of Minnesota College of Pharmacy, Minneapolis, MN, United States; ^2^Department of Psychiatry, University of Minnesota Medical School, Minneapolis, MN, United States; ^3^University of Minnesota Supercomputing Institute, Minneapolis, MN, United States; ^4^Minneapolis Veterans Affairs Health Care System, Minneapolis, MN, United States; ^5^Defense Veterans Brain Injury Center, Minneapolis, MN, United States

**Keywords:** PTSD, meditation, MBSR, treatment response, epigenetics, DNA methylation, FKBP5, SLC6A4

In the original article, there was an error. We have identified four sentences where the words “increase” and “decrease” were mistakenly transposed. These four transpositions do not affect the integrity of the data and were the result of an error in translating the sign of the effect sizes from analyses to text. As a result of correcting these four transpositions, two sentences in the discussion contextualizing direction of change required modification.

A correction has been made to ***the Abstract sentences #9 and 11, the Results subsection*** ***FKBP5 Change from Before to after MBSR Treatment sentence #1, the Discussion*** ***subsection FKBP5 sentence paragraph 1sentence #1, paragraph 2 sentence #5, and*** ***paragraph 3 sentence #2*.**

**Abstract sentence #9:** There was a significant time x responder group interaction for methylation in *FKBP5* intron 7 bin 2 [*F*_(1, 19)_ = 7.492, p = 0.013] whereby responders had an increase in methylation and non-responders had a decrease in methylation from before to after treatment in this region.

**Abstract sentence #11:** Increases in *FKBP5* methylation after treatment in responders as compared to decreases in non-responders suggest that effective meditation intervention may be associated with stress-related pathways at the molecular level.

**Results>Methylation of FKBP5>Change from Before to after MBSR Treatment>sentence #1:** There was a significant time x responder group interaction for methylation in *FKBP5* intron 7 bin 2 [*F*_(1, 19)_ = 7.492, *p* = 0.013, Bonferroni adjusted *p* = 0.052; Figure 4 and Supplemental Table 2] whereby responders had an increase in methylation and non-responders had a decrease in methylation from before to after treatment in this region. **Discussion>FKBP5>sentence #1:** Analyses of *FKBP5* revealed increases in intron 7 methylation after treatment in responders while non-responders had decreases in methylation within a specific *FKBP5* CpG site of bin 2.

**Discussion>FKBP5>Paragraph #2>sentence #4:** Additionally, consistent with our findings is that despite examining a different regulator region of *FKBP5* than our study (exon 1 promoter vs. intron 7 GRE in our study) both of these investigations showed that changes in methylation were related to better symptom response with corresponding measures of increased gene expression identified in the study of PTSD response to exposure therapy (24).

**Discussion>FKBP5>Paragraph #3>sentence #2:** Collectively our findings as well as those of others are consistent with the hypothesis that non-pharmacologic interventions may facilitate stress reduction through the regulation of FKBP5 to feedback on glucocorticoid hyperactivity to reduce stress.

The authors apologize for this error and state that this does not change the scientific conclusions of the article in any way. The original article has been updated.

